# A Different Vision: Centering Love Not Punishment for Families Affected by Substance Use

**DOI:** 10.1007/s10995-023-03843-w

**Published:** 2023-11-13

**Authors:** Kimá Joy Taylor

**Affiliations:** Anka Consulting LLC, Silver Spring, MD USA

**Keywords:** Maternal child health, Wellness: substance use, Racial and health equity

## Abstract

Improving maternal and child outcomes requires us to understand and deconstruct our country’s historically punitive policies toward pregnant and parenting people who use drugs. We also must build a new system that centers wellness in partnership with individuals directly affected by these policies. From a maternal and child health (MCH) perspective, wellness is defined as parent-infant dyads living in supportive, preserved, and loving families with access to the resources needed for optimal health. To achieve wellness and positive outcomes, all individuals must have equitable access to a full continuum of culturally and linguistically effective, geographically available, evidence-informed, non-punitive, and welcoming health and social services that prioritize family preservation. In addition, to attain transformative and equitable outcomes, advocates for families affected by substance use must focus on implementing and evaluating services and continuously monitoring disaggregated data to ensure inequities are eliminated.

## Significance

Conversations about the opioid crisis often ignore the needs of pregnant and parenting people who use drugs. This paper seeks to address this gap.

## Introduction

I work in many maternal health spaces where policymakers often miss the forest for the trees. After a meeting with policymakers, where women’s health advocates were pursuing a narrow health policy concession, a colleague asked me, “don’t they want us [Black women] to be well?” My soul hurt because I realized that others in the room had not even considered that goal. My colleague was referring to overall wellness, the ability to strive for our best physical and emotional health, a goal we all want for those we love. For this commentary, I would like us to hold the well-being of the parent-child dyad—including for pregnant and parenting people who use drugs—as a focal point and a way to improve health outcomes and preserve families.

Why is centering physical and behavioral wellness for families affected by substance use so important? As you have read in the previous articles in this journal supplement, the United States is experiencing a long-running MCH crisis, with disproportionately adverse outcomes for Black and Indigenous pregnant people. Between 2017 and 2020, overdose rates during pregnancy and the postpartum period nearly doubled (Bruzelius & Martins, 2022). Data from maternal mortality review committees show that behavioral health conditions are one of the leading preventable causes of maternal mortality (Trost et al., 2022). However, these statistics do not comprehensively reflect the maternal morbidity caused by behavioral health conditions among those who have been historically and systematically marginalized. For instance, data show that Black women experience perinatal mood disorders at twice the rate of white women and are less likely to receive treatment (Bruzelius & Martins, 2022).

The reality is that we do not have all the disaggregated data we need to get a complete picture of the impact and inequities of behavioral health conditions, particularly as they relate to pregnant and parenting people who use drugs. We do, however, have enough information to recognize that society has a problem. Despite compelling, albeit incomplete, data, many federal, state, and local policies that seek to improve maternal mortality and morbidity rates discuss behavioral health as an afterthought. Pregnant and parenting people with current or past drug use often face discrimination, stigma, criminal-legal involvement, family disruption, and a dangerous lack of access to evidence-informed, culturally, and linguistically effective care (Weber et al., 2021). Suppose our society truly wishes to eliminate health inequities and improve maternal mortality rates. In that case, non-punitive responses must be integrated into all programs, practices, and conversations for pregnant people who use drugs.

### What Would a Wellness Framework Look Like?

A wellness framework requires equitable access to and positive outcomes from a full continuum of physical health care, mental health care, substance use disorder (SUD) treatment, and social services that seek to preserve and support families. These services should be welcoming, culturally and linguistically effective, geographically accessible, community-based, evidence-informed, and non-punitive. This type of comprehensive continuum of care is rare for many pregnant people, as demonstrated by the country’s abysmal maternal mortality rate. But, because of historical policies, practices, and programs, it is even less accessible for pregnant and parenting people with SUD.

Wellness is not a “new” vision for those who love their families, for parents, including those with a substance use disorder, who are raising their children and centering love and well-being to the best of their ability, despite societal and structural racism, stigma, and punitive responses. Unfortunately, centering wellness often comes at the cost of parents avoiding the care and support systems that *all* parents need because these systems have been historically harmful in ways that affect them and their loved ones’ physical and mental health and may even lead to family separation.

### How Can We Move Forward?

Ideally, society would deconstruct and rebuild our maternal health system by centering the parent-infant dyad with embedded policies and practices that require equitable outcomes. Unfortunately, this is unlikely in our current environment. Therefore, I offer some achievable steps that only represent some of the possibilities for the change we need but can move society forward if thoughtfully implemented.

#### Partner with Impacted Populations

Future work addressing MCH inequities must include diverse voices of pregnant and parenting people with SUD, including those affected by the family regulatory system. Policymakers should not focus solely on individuals in recovery but also talk with individuals who currently use drugs. These voices must be integral to the process, not as token participants but as true partners. People who use drugs can help shape responses and assess the effectiveness of these responses to achieve equitable outcomes. This work requires teaching policymakers, practitioners, systems officials, researchers, and others how to nonjudgmentally embrace the input of community representatives and how to develop, value, and understand community-led initiatives and desired outcomes. The work could include participatory budgeting in which community input helps determine how money is allocated to achieve equitable wellness aims.

#### Fund and Support Policy Implementation and Evaluation

Policies may sound effective and equitable, but implementation and evaluation are where the rubber hits the (often long) road. Many researchers and policymakers have floated reasons for the discrepancy between policy intent and outcomes for different populations. But this discrepancy speaks to a need for more attention to the goal. When implementing new policies and laws, will the policymakers provide funding and time to work in partnership with all parties and do real-time evaluations? Do we have the funding and capacity to collect, disaggregate, and analyze data for all communities demonstrating progress toward eliminating inequities and when we may need to change course? I offer the following three policy examples that will benefit from this approach to implementation.

### Medicaid One-Year Postpartum Extension

Medicaid postpartum extension is a significant achievement, and its rapid uptake among states is encouraging. Now, we must ensure that pregnant and parenting people with SUD achieve equitable access to a full continuum of culturally effective SUD treatment services in the postpartum period, a time when people often resume substance use. To determine the true effectiveness of this policy, we must not only disaggregate MCH outcomes based on race, ethnicity, sexual orientation, gender, and other intersectional identities but also include outcomes for postpartum people who use drugs.

### Screening for Prenatal Drug Use

Many healthcare providers and hospitals implement confusing drug screening policies for pregnant patients that often demonstrate racial bias. We must prioritize voluntary, evidence-based verbal screening rather than requiring urine tests for pregnant people suspected of drug use and child welfare referrals. Notably, a urine test is not a clinical test to diagnose SUD, nor can it measure parenting ability or the existence of child abuse. We must ask, are there equitable, positive family preservation outcomes associated with screening policies? If not, why not? Where is there a breakdown in the system, and how can training, program, policy, or practice change eliminate inequities in screening practices?

### Removal of the X Waiver Requirement

Federal policymakers have slowly expanded the ability of providers to prescribe buprenorphine to people who use drugs by removing the eight-hour provider certification requirement (the “X waiver”) in 2022, which resulted in treatment expansion for thousands of people. However, racial and ethnic disparities persist regarding who can access buprenorphine (Schiff et al., 2020). Research in Massachusetts demonstrated that Black Non-Hispanic and Hispanic women were less likely to receive buprenorphine for opioid use disorder, even though this medication is considered the gold standard for pregnant people who use drugs. Like the Medicaid postpartum extension, the X waiver policy change is relatively new. Measuring its true impact on access to treatment for pregnant and postpartum people will require disaggregating data based on race, ethnicity, sexual orientation, gender, and other intersectional identities.

#### Remove Roadblocks in Other Systems

SUD treatment and medical care alone are not enough to improve the health and well-being of the parent-infant dyad. When creating holistic, wellness-centered care, partners should help remove barriers to the social determinants of health, such as food assistance, housing, employment, and lifesaving basic human services for people with SUD. Furthermore, as mentioned in other articles in this supplement, the family regulatory system can outsize the impact on health and well-being, potentially causing family disruption and separation. Not surprisingly, parents have stated that losing their children led them to increase their drug use (Lamonica & Boeri, 2020). The family regulatory system and the systems related to the social determinants of health must be responsive to the needs of pregnant and parenting people who use drugs through non-punitive and welcoming approaches.

#### Educate, Train, and Diversify All Levels of Staffing

People who deliver services to pregnant people and parents who currently use drugs or are in recovery often lack the knowledge, attitudes, and diversity to center wellness and improve outcomes effectively.

### Education

Most health professional training programs provide inadequate instruction on SUD or the continuum of care required to improve outcomes for people who use drugs. Less than a third of medical schools offer training in addiction medicine (Harris, 2023), and less than 5% of accredited social work schools have one or more required courses in addiction (Wilkey et al., 2013). It therefore makes sense that many providers feel even less equipped to offer evidence-informed SUD services to pregnant people. To address this problem, all health professional training programs must be held accountable for closing this educational gap. In addition, training for all system staff and administrators is deeply needed. We often solely focus on provider training on substance use, implicit and explicit bias, and stigma. Although this training is imperative, it does not address the fact that a patient will engage and interact with other people (e.g., administrative and clinical staff in prenatal care settings) who may positively or negatively affect their health care experience and even their desire to remain in care. SUD education must be provided for these individuals as well.

### Training to Provide Respectful Care

A long history of mental health and substance use stigma has led many staff working in health care systems to believe it is acceptable to treat patients who use drugs disrespectfully, including pregnant patients. Repeated studies on patient care reveal stories of cruelty, which can lead to women avoiding prenatal and other physical and mental health services that could improve outcomes and overall wellness for parents and infants (Weber et al., 2021). Respectful maternity care is an approach that encourages shifting practice, such as being responsive to patient trauma, to reduce inequities in birth outcomes (Scott et al., 2024).

### Diversification of the Behavioral Health Workforce

Staff in health care systems, including those in substance use treatment and obstetrics, must reflect the diversity of the people they serve. They must represent a range of racial and ethnic identities, people with lived experience, and other intersectional identities. The severe shortage of behavioral health providers is compounding the problem of lack of diversity. In some instances, a patient with a racially concordant provider can improve patient satisfaction and outcomes (Shen et al., 2018). Similarly, including peers on the substance use service team can increase patient engagement, satisfaction, and retention (Eddie et al., 2019). While providers and systems should understand each person’s preferences to tailor treatment, studies demonstrate a need to expand and diversify the ranks of those who provide care to pregnant and parenting people who use drugs (NASHP, 2021).

#### Hold Systems Accountable

While eradicating racism and eliminating bias may be long-term goals, society can presently hold systems and people accountable for creating welcoming, nonjudgmental, culturally, linguistically effective, and accessible care. Many healthcare systems default to referring pregnant and postpartum people who use drugs to the family regulatory system (Maclean et al., 2022). If entities refuse to offer or refer clients to a full continuum of evidence-informed substance use services, including harm reduction and medications, these entities should lose their licensing and funding. Patients have the right to manage their care and reject certain services; however, the days when treatment programs could refuse or could choose whom to provide medications for opioid use disorder must end.

Organizations must collect, disaggregate, and analyze data from all health and social service providers to glean whether all population groups have equitable access to and outcomes from these services. When inequities are identified, providers and systems must create plans to eliminate inequities. If the problems persist, systems should be fined, and if the level of inequities is egregious, they should lose funding or accreditation.

## Conclusion

Re-envisioning current systems to include and address the well-being and specific needs of diverse pregnant people who use drugs will help to equitably improve maternal morbidity and mortality. Policies, practices, and programs should learn how to do this and be held accountable for providing nonjudgmental, non-punitive, evidence-informed, culturally, and linguistically effective care. Substance use, mental and physical health treatment, and social services must be integrated to center well-being and family preservation. This new vision will require society to educate advocates, policymakers, providers, payers, and other system partners to abandon long-held punishment paradigms and embrace the science of substance use care and well-being. This vision will also require providers to partner with pregnant and parenting people impacted by SUD. Ultimately, this wellness vision will not only improve health outcomes and family preservation for families impacted by SUD but for *all* pregnant and parenting people.

“*Making the decision to have a child—it is momentous. It is to decide forever to have your heart go walking around outside your body.”* Elizabeth Stone.

I am a parent, and this quote resonates with me. Our society pretends that pregnant and parenting people who use drugs do not deeply care for their children in the same way as others. We enact policies that make this already difficult job of parenting much harder and even rips the maternal-child bond forever for some people.

Throughout our country’s history, we have employed a punishment system that leads to inequitable outcomes for the parent-infant dyad, especially for Black, Indigenous, and other families of color impacted by substance use. We can do better. As a society, we can and must turn toward a wellness paradigm. Centering the idea that proactively seeking health and wellness in its many forms is something *all of us* can do in concert and partnership with others so that parent-infant dyads, including those affected by drugs, have equitable access to services and positive outcomes from access to the basic human life necessities. We must ensure that all people have the health and social support they need to thrive together as a preserved, loving family. Parenting is hard work, but it is momentous and wonderful. I have yet to meet a parent who cannot use support, although it may be hard for them to ask. I have also met very few parents who do not love their children, and this is equally true for pregnant people who use drugs. As a society, we can support this deep parental love and make it easier for them and all pregnant people to ask for the support needed to preserve parent and infant well-being and the family as a whole.

## Data Availability

Not applicable.
